# The effects of empathy skills training on nursing students’ empathy and attitudes toward elderly people

**DOI:** 10.1186/s12909-018-1297-9

**Published:** 2018-08-15

**Authors:** Sakineh Gholamzadeh, Maryam Khastavaneh, Zahra Khademian, Soraya Ghadakpour

**Affiliations:** 1 Community-Based Psychiatric Care Research Center, Shiraz University of Medical Sciences , Shiraz, Iran; 2 School of Nursing and Midwifery, Shiraz University of Medical Sciences, Shiraz, Iran

**Keywords:** Attitude, Aging, Nursing students, Empathy, Education

## Abstract

**Background:**

Nursing students’ empathy and positive attitudes toward elderly people could help provide improved elderly care in their future practice. This study aimed to investigate the effects of empathy skills training on nursing students’ empathy and attitudes toward elderly people.

**Methods:**

This quasi-experimental study was conducted in Yasuj, Iran in 2014. The sample consisted of 63 students at Hazrat Zeinab Nursing and Midwifery School who were randomly divided into a control (*n* = 31) and an intervention group (*n* = 32). The intervention group attended an eight-hour workshop on empathy skills that was presented through lectures, demonstration, group discussions, scenarios, and questioning. The data were collected using the Persian versions of Kogan’s Attitudes towards Old People Scale and Jefferson Scale of Physician Empathy-Health Professionals Version. Then, the data were entered into the SPSS software, version 19 and were analyzed using descriptive statistics, chi-square test, t-test, and repeated measures analysis of variance.

**Results:**

The results showed that the empathy skills training program had a significant impact on the students’ mean scores of empathy and attitudes toward elderly people (*p* < 0.001). The intervention group’s mean score of empathy increased from 77.8 (SD = 10.7) before the intervention to 86 (SD = 7.3) immediately after that and 85.2 (SD = 8.9) 2 months later. Their mean score of attitude also increased from 110.8 (SD = 10.9) before the intervention to 155.2 (SD = 23.4) immediately after the intervention and 158.6 (SD = 23.2) 2 months later. Additionally, the empathy and attitude scores of the intervention group were significantly higher than those for control group immediately and 2 months after the intervention.

**Conclusions:**

Empathy skills training improved the nursing students’ empathy and attitudes towards elderly people. Therefore, empathy training is recommended to be incorporated into the undergraduate nursing curriculum.

## Background

In the present century, as a result of rise in life expectancy, old age is becoming an important worldwide phenomenon. World Health Organization (WHO) defined elderly people as individuals aged 60 years and above. According to the statistics released by WHO, the population of elderly people aged 60 years and above in the world was 900 million in 2015, which has been estimated to reach 1.2 billion by 2025 and 2 billion by 2050 [1]. In Iran, the elderly population increased by 4 times during 1967 to 2011 [2]. In 2011, individuals older than 65 years comprised 5.7% of Iran’s population [3]. It has been estimated that nearly 30% of the Iranian population will be 60 years old and above by 2050 [1].

Elderly people have various healthcare needs. As a result of the physiological changes that accompany old age, elderly individuals comprise the most vulnerable group to various diseases. For instance, three out of four Americans aged 65 years and above suffered from multiple chronic conditions, and 93% of Medicare was spent for individuals with multiple chronic conditions [4]. Some chronic diseases, in turn, lead to disablement in activities and frequent hospitalizations [5]. Other health problems, such as depression and cognitive impairment, may also interfere with elderly individuals’ communication abilities and cause other special healthcare needs. The prevalence of cognitive impairment was 7.85% in an Iranian elderly population [6]**.**

Rapid growth of the elderly population and their vulnerability to various health disorders lead *to* increased number of elderly patients needing healthcare services. Nurses, as members of the healthcare team, are becoming increasingly responsible for caring for elderly people. Additionally, they have a key role in ensuring patients’ safety and acting on their behalf [7]. Elderly care may be affected by various factors, including nurses’ attitudes towards elderly people. Hence, nurses’ attitudes towards elderly people could determine the quality of therapeutic interaction between nurses and elderly patients [8] as well as nurses’ willingness to care for these patients [9]. According to studies, some nurses and nursing students had negative attitudes towards elderly individuals [10–12] and were not willing to consider elderly care as a part of their future jobs [13]. A cross-sectional Iranian study also indicated that 54.3% of nurses had negative attitudes towards older adults [10]. Thus, measures have to be taken to improve nurses’ attitudes towards elderly care, especially in health educational settings.

The proper relationship between healthcare providers and elderly people is important in achieving the goals of the care plan [14]. Nurses, compared to other members of the healthcare team, spend more time interacting with patients. Therefore, it is especially important to train them in establishing positive relationships with patients [15].

Empathy is a basic competency of helping relationship and an integral component of person-centered care. Person-centered care, in turn, improves the quality of care and patients’ outcomes [14, 16]. Empathy towards patients increased patients’ satisfaction and reduced their distress [17]. Empathy has been defined as a cognitive attribute involving an understanding of patients’ experiences, concerns, and perspectives together with the ability to communicate this understanding and the intention to help [18]. Iranian hospitalized elderly patients reported their needs for an empathetic understanding [19]. On the other hand, a qualitative study showed that nurse assistance communication with elderly people in home care was mostly task-oriented and that person-oriented communication had to be promoted in nurse-elderly communication [20]. In addition, it has been reported that nursing students’ empathy towards elderly people somewhat decreased during academic education [21, 22]. Empathy is a teachable competency [23], which is particularly important for students in the last years of their education. Therefore, it is important to investigate evidence-based interventions to improve nursing students’ empathy and attitudes towards elderly people.

A systematic review reported that only a few studies on healthcare students investigated the effect of workshops on students’ attitudes towards elderly people, and that some of these interventional studies lacked control groups or randomization [24]. Therefore, further studies have to be conducted in this domain to examine the effects of workshops on nursing students’ attitudes towards elderly people. It should be noted that traditional nursing education lacked the appropriate teaching methods to train nursing students about empathy skills. Hence, it is essential to find effective ways to empower nursing students with these skills [16]. Evidence has revealed the beneficial effects of training nursing students regarding non-technical skills on their attitudes [25, 26]. However, there is not sufficient evidence to determine whether empathy-building workshop can improve nursing students’ empathy and attitudes towards elderly people. Therefore, the present study aims to explore the effects of training nursing students regarding empathy skills on their empathy and attitudes towards elderly individuals (≥ 60 years old). The content of this training workshop and the study population (third- and fourth-year nursing students) make the study unique among the studies with empathy-building interventions. Moreover, it was previously reported that a short-term intervention improved health-care students’ empathy towards elderly people, while this improvement declined over time [27]. Therefore, the present study examined the effects of empathy skills training both immediately and 2 months after the intervention.

## Methods

### Design and sample

The present quasi-experimental study with a pretest/posttest control design was conducted in 2014. All third- and fourth-year nursing students at Hazrat Zeinab Nursing and Midwifery School of Yasuj, Iran were included in the study. Then, the 70 students were randomly divided into a control and an intervention group through block randomization. It should be noted that during the study, four students from the control group and three students from the intervention group were excluded due to unwillingness to participate, and the study was continued with 63 students (32 in the intervention group and 31 in the control group).

The inclusion criteria of the study were willingness to participate, being a third- or fourth-year nursing student, and not having taken any empathy courses in the past 6 months. In case the students were unwilling to continue participation in the study or were participating in another educational program at the same time, they were excluded.

### Data collection and intervention

The study data were collected using the Persian versions of Kogan’s Attitudes towards Old People Scale (KAOP) and Jefferson Scale of Physician Empathy-Health Professionals Version (JSPE-HP). JSPE-HP was a 20-item questionnaire, all of which addressed the respondents’ level of empathy with patients. The items were answered based on a 5-point Likert scale ranging from 1 (strongly disagree) to 5 (strongly agree). The scores could range from 20 to 100, with higher scores indicating greater empathy with elderly patients. The scores of 77 and below were considered poor, those between 78 and 88 were considered moderate, and scores above 88 were considered good. The validity and reliability of the Persian version of the questionnaire were evaluated in Hashemipour’s study (2012) in Iran. Accordingly, Cronbach’s alpha coefficient of the questionnaire was 0.83 [28].

KAOP contained 34 positive and negative items; 17 items about positive attitudes and 17 items about negative attitudes. It evaluated the respondents’ attitudes towards elderly people based on a 6-point Likert scale ranging from strongly disagree (1) to strongly agree (6). The negative items were scored reversely, such a way that the scores ranged from strongly agree (1) to strongly disagree (6). The total score of the questionnaire could range from 34 to 204. The scores of 111 and below were considered poor, those between 112 and 118 were considered moderate, and scores above 118 were considered good (20). Several studies have indicated KAOP as a reliable and valid instrument for evaluating individuals’ attitudes towards elderly individuals. Cronbach’s alpha coefficient of the whole scale (positive and negative items) has been reported to vary from 0.79 to 0.87 [29].

The students in both groups were asked to complete the demographic data questionnaire, JSPE-HP, and KAOP. The intervention consisted of an eight-hour workshop on empathy skills that was held at the college for 2 days. The content of the workshop was designed by the researchers and reviewed and revised by some of the college professors. The workshop was mainly based on constructivist learning theory. This theory considers the learners active in the construction of knowledge through real life experiences. Social and cultural aspects of learning and learners’ ideas about the learning topic are emphasized, as well [30]. All students in the intervention group participated in the same workshop. The students were informed about the date of the workshop in advance. The content was presented through lectures, role play, group discussions, scenarios, clip presentation, and questioning. In the workshop, the students were informed about the epidemiological transition for elderly people, physiological changes in elderly people, professional relationship with elderly people, self-awareness, and definition and examples of empathy towards patients. The topics, learning objectives, activities, and timetable have been presented in Table 1. The lectures on self-awareness and empathy were presented by experienced psychology professors, and the rest of the material was presented by one of the researchers. The students in both intervention and control groups completed the questionnaires before, immediately after, and 2 months after the intervention.

**Table 1:** Empathy-building workshop program

Topics	Learning objectives (At the end of this workshop the students will be able to …)	Learning activities	Approximate time (min)
Introduction	… describe the importance and objectives of empathy-building workshop	Lecture	15
Epidemiology of aging	… describe epidemiology of aging	Lecture	20
Physiological and psychological changes of aging	… explain physiological and psychological changes of aging	Lecture, question and answer	60
Common barriers to communication in older adults	… explain barriers to communication in older adults	Lecture, group discussion, Clip presentation	30
Exercise on communication	… analyze challenges and their own experiences in communication with elderly people	- Three scenarios were presented: - The students were asked to identify and discuss the challenges in the scenarios - Encouraged students to discuss their experiences of similar situations with elderly people - Giving feedback and summing up	40
Interactions between healthcare professionals and older patients	… interact professionally with older patients in a simulated situation	Role play on listening skills, lecture, group discussion	60
Definition of self-awareness and its importance	... discuss self-awareness and its importance	Lecture and group discussion	30
Exercise on self-awareness	… identify their own attitudes toward aging and its impact on their communication with elder patients	-The students were asked to identify their feelings and attitudes toward aging and its impact on their communication with elderly patients -Giving feedback and summing up	45
Empathy skills, understanding, listening, accepting others, etc.	… explain empathy skills and their importance	Lecture and group discussion	90
Exercise on empathy skills	…discuss empathetic behaviors and lack of empathy in nurse-patient relationship ….identify their own barriers and abilities to show empathy toward older adults	- Presenting one scenario and two short clips about empathy with elderly patients -Asking students to discuss the scenario within their groups and identify empathic or lack of empathic behaviors. - Giving feedback and summing up	90

### Data analysis

The collected data were entered into the SPSS statistical software, version 19 and were analyzed using descriptive statistics, chi-square test, independent t-test, Mann-Whitney, and repeated measures ANOVA. The effect sizes of 0.2, 0.5, and 0.8 were considered small, medium, and large, respectively [31].

## Results

The majority of the participants in both the control (53.1%) and the intervention group (58.1%) were female, with the mean age of 22.7 (SD = 1.62) years. Based on the results of chi-square test and independent t-test, there were no statistically significant differences (*p* > 0.05) between the two groups in terms of demographic characteristics. Also, there was no difference between two groups regarding the study variables before the intervention (Tables 2 and 3).

**Table 2:** Baseline comparison of the two groups regarding the frequency distribution of the qualitative contextual characteristics^a^

Group	Control (*n* = 31)	Intervention (*n* = 32)	Total	*P*-value
Variables	Frequency (%)	Frequency (%)	Frequency (%)	
Gender				
Female	17 (53.1)	18 (58.1)	35 (55.6)	0.693
Male	15 (46.9)	13 (41.9)	28 (44.4)	
Academic year				
Year 3	17 (53.1)	9 (29)	26 (41.3)	0.052
Year 4	15 (46.9)	22 (71)	37 (58.7)	
Living with an elderly individual				
Yes	20 (62.5)	17 (54.8)	37 (58.7)	0.537
No	12 (37.5)	14 (45.2)	26 (41.3)	

^a^Chi-square test

**Table 3:** Baseline comparison of the two groups regarding the study variables^a^

Group	Control (*n* = 31)	Intervention (*n* = 32)	Total	*P*-value
Variables	Mean (±SD)	Mean (±SD)	Mean (±SD)	
Age	22.8 (±2.17)	22.5 (±1.07)	22.7 (±1.62)	0.521
Attitude towards elderly people	110 (±12.3)	110.8 (±10.9)	110.4 (±11.5)	0.775
Empathy	78.6 (±10.1)	77.8 (±10.7)	78.1 (±10.3)	0.77

^a^Independent t-test

The pretest total mean score of the students’ attitude was 110.4 (SD = 11.5), which indicates the students’ poor attitudes towards elderly people. In addition, the pretest total mean score of empathy was 78.1 (SD = 10.3), which indicates the nursing students’ moderate level of empathy (Table 3).

In terms of the mean scores of attitude, there was a significant difference between the intervention and control groups immediately (*p* < 0.001) and 2 months after the intervention (*p* < 0.001). There were also significant differences in both intervention (*p* < 0.001) and control groups (*p* < 0.001) over time. The effect size for attitude scores was medium in the intervention and small in the control group (0.71 vs. 0.36) (Table 4). The intervention group’s mean score of attitude rose from 110.8 (SD = 10.9) before the intervention to 155.2 (SD = 23.4) immediately after that. On the other hand, the control group’s mean score of attitude rose from 110 (SD = 12.3) before the intervention to 126.6 (SD = 13) immediately after that. Two months after the intervention, the mean score of attitude was 158.6 (SD = 23.2) in the intervention group and 128.4 (SD = 13.3) in the control group (Table 4). The results of Mann-Whitney test revealed that the posttest/pretest mean difference of the attitude score was significantly higher in the intervention group compared to the control group immediately after (44.4 (SD = 27.09) vs. 16.6 (SD = 16.6), *p* < 0.001) and 2 months after the intervention (47.8 (SD = 26.7) vs. 18.4 (SD = 17), *p* < 0.001). These indicate the effectiveness of the intervention in the intervention group.

**Table 4:** Comparison of the two groups regarding the mean scores of attitude and empathy in three time points

Variables	Group	Before the intervention	Immediately after the intervention	Two months later	F (*p*-value)	Effect size	Power
		Mean (± SD)	Mean (± SD)	Mean (± SD)			
Attitude	Intervention	110.8 (±10.9)	155.2 (±23.4)	158.6 (±23.2)	76.4 **(< 0. 001)**^**a**^	0.71	0.100
	Control	110 (±12.3)	126.6 (±13)	128.4 (±13.3)	33.6 **(< 0.001)**^**a**^	0.53	0.100
	P-value	0.78^**b**^	**< 0.001** ^**c**^	**< 0.001** ^**c**^			
	Power	0.50	0.100	0.100			
Empathy	Intervention	77.8 (±10.7)	86 (±7.3)	85.2 (±8.9)	17.3 **(< 0.001)**^**a**^	0.36	0.99
	Control	78.6 (±10.1)	76.7 (±11.9)	77.5 (±11.3)	1.1 (0.32)^**a**^	0.03	0.19
	P-value	0.77^**b**^	**< 0.001** ^**c**^	**0.004** ^**b**^			
	Power	0.30	0.97	0.86			

^**a**^Repeated measures analysis of variance

^**b**^Independent t-test

^**c**^Mann-Whitney test

The bold numbers are significant *p*-values

In terms of the mean score of empathy, there was a significant difference between the intervention and control groups immediately (*p* < 0.001) and 2 months after the intervention (*p* = 0.004). Moreover, there were significant differences in this regard in the intervention group over time (*p* < 0.001). The effect size of the intervention group’s empathy score was small (0.36). The intervention group’s mean score of empathy rose from 77.8 (SD = 10.6) before the intervention to 86 (SD = 7.3) after that (an 8.2-score increase). However, the control group’s mean score of empathy declined from 78.6 (SD = 10.1) before the intervention to 76.7 (SD = 11.9) after that (a 1.91-score decrease). Two months after the intervention, the intervention group’s mean score of empathy was 85.2 (SD = 8.9) with a 7.4-score increase compared to before the intervention. On the other hand, the control group’s mean score of empathy was 77.5 (SD = 11.3) with a 1.1-point decrease. Moreover, the intervention group’s mean score of empathy decreased by 0.8 points 2 months after the intervention compared to immediately after that, but the difference was not statistically significant (*p* = 0.33). However, the change was not significant in the control group (*p* = 0.32) (Table 4). 

## Discussion

The results of the current study indicated that the empathy skills training improved the students’ scores of empathy and attitudes towards elderly people. The effect size was small for empathy scores and medium for attitude scores. These indicate that the intervention was somewhat effective in empathy scores and moderately effective in attitude scores. In the workshop, the students were encouraged to discuss aging as well as their past experiences and attitudes towards elderly individuals and to identify their abilities and barriers against showing empathy towards older adults. It seems that these learning contents alongside encouraging active learning and valuing students’ ideas and experiences on the topics that were rooted in the constructivism learning theory contributed to these findings. Similarly, a meta-analysis revealed that constructivist learning approaches were moderately effective in learners’ attitudes towards learning subjects [32]. Another study showed that nursing students used different dynamic learning strategies in their own learning process [33]. The findings of the present study are in agreement with those of other studies that used different interventions. For example, Chen et al. reported that nursing students’ empathy and attitudes towards elderly people improved after they played the role of an older adult in a simulation game [34]. In the same line, an innovative bonding program, including workshop and opportunity to work with elderly people in the community, improved nursing students’ attitudes regarding elderly individuals [35]. On the contrary to these findings, an Iranian study demonstrated that an elderly care plan did not improve nursing students’ attitudes towards elderly people [36].

While empathy-building interventions could improve nursing students’ attitudes toward disabled patients [37], review of the literature revealed no studies indicating the effectiveness of empathy skills training in students’ attitudes towards elderly people. Therefore, the present study findings could not be compared to those of other studies. Hence, these findings may provide a novel piece of knowledge in this field. Accordingly, empathy-oriented workshops are recommended to improve nursing students’ attitudes and empathy towards elderly people. This will help prepare them to communicate effectively with elderly individuals in their future professional performance.

Although the change in the control group’s mean score of attitudes towards elderly people was significant, it was significantly lower than that of the intervention group. The change in the control group’s attitude scores might be attributed to the effect of their teachers and other nurses as role models or other factors out of this study scope.

This study did not aim to investigate the effect of the intervention on the students’ willingness to work with elderly patients. Thus, it could not be concluded that the students’ willingness to care for elderly people increased. This is recommended to be examined in future studies.

At the beginning of the present study, all nursing students reported poor attitudes towards elderly people, which have been reported by other studies, as well. Tabiei et al. found that Iranian nursing students had an unsatisfactory attitude towards care for elderly patients with cardiovascular diseases [38]. Similarly, Sanagoo et al. concluded that adolescents, young adults, and young college students had the most negative attitudes towards elderly people [29]. In addition, an Iranian study showed that third-year college students had neutral attitudes and first-year ones had negative attitudes towards elderly individuals [39]. In contrast, other studies found that nurses and nursing students had satisfactory attitudes towards elderly people [40, 41]. These discrepancies might be attributed to differences in sample sizes, research populations, or even cultural and social differences. Cultural aspects should be deliberately taken into account in studies on attitudes towards and communication with elderly people. A study on nurses and nursing students in six different countries showed different attitudes towards older people [42]. In eastern countries, respect for elderly individuals is a cultural value and older persons are considered as wise people. However, Huang’s study indicated that undergraduate students from western countries had more favorable attitudes towards older adults in comparison to those from eastern countries [43].

## Limitations

The students in the intervention group were asked not to communicate with other students about the workshop until the end of study. In addition, the intervention group students were not provided with any printed materials to avoid data contamination between the two groups. However, the possibility of data contamination could not be neglected. Moreover, the mid-term effects of the intervention were assessed after 2 months. Data collection in more extended times could assess the long-term effects of the intervention. Furthermore, self-assessed empathy and attitude scores of the nursing students were used in this study. More objective assessments, such as students’ performance in empathy skills and their willingness to care for elderly people, could reveal study outcomes and students’ learning transfer in practice more efficiently. Another study limitation was related to the challenges of using KAOP for data collection since it has been suggested that the questionnaire needs updating [44].

## Conclusion

The results of this study showed that development and execution of a unique 8-h workshop on empathy significantly improved the intervention group’s scores of empathy and attitude toward elderly people. Empathy is a teachable skill and policymakers at nursing education institutions are recommended to use the results of the present study and incorporate empathy skills training into undergraduate nursing education. This may serve to achieve two goals; reinforcing students’ empathy towards elderly people and improving their attitudes toward older adults.

## Abbreviations

ANOVA: Analysis of Variances
JSPE-HP: Jefferson Scale of Physician Empathy-Health Professionals Version
KAOP: Kogan’s Attitudes towards Old People Scale
WHO: World Health Organization
